# Indents

**Published:** 1910-11-15

**Authors:** 


### INDENTS.

**☞Brief Articles of Technical or Scientific Value will receive notice and comment. Contributions invited.**

*Plating Plant.* Some sort of a gold plating plant seems an important auxillairy to the dental equipment of to-day.

A simple one is made up of a glass pickle jar of convenient size with a cover to prevent evaporation and exclude dust containing a fluid composed of 30 grains Chloride of Gold, Merck, U. S. P.) 60 grains Cyanide of Potassium, and ½ pint of distilled Water, operated by a single cell Sampson battery. A piece of pure gold is attached to the carbon wire and suspended in the fluid, while the atricle to be plated is attached to the zinc wire and likewise suspended, care being taken that the gold and the article to be plated do not come in contact while in the solution.

In place of the Sampson cell the wires may be connected with the ordinary electric lighting supply, in which case the current is run through a series of lamps to reduce it.

In the latter case, the piece to be plated is left in the solution perhaps five minutes, when it is taken out and polished; this process is repeated several times according to the amount of plate desired. Placing the jar of solution in a hot water bath facilitates the work.—*The Journal.*

*Suction Plate for a Lower Denture.* I do not know if the following method is new or not, but I have never seen it described in dental text-books or journals, and wish my fellow practitioners to know of it, for to me it has been a great boon.

To make a suction plate for a very flat or hard lower alveolar ridge, proceed as follows: Immerse *the model* for one hour in an alum solution—one part alum (free from iron), eight parts water; this will toughen the plaster. At any time before packing the rubber, cut in the plaster model of a wedge-shaped groove with the thin edge pointing outward; I use a blunt instrument first and make a groove two-eights of an inch inside the outer limits of the plate line all around the lingual, labial and buccal surfaces, and join them at the heel of the model. Next deepen the groove gradually with a wedge-shaped sharp instrument (I use Dr. R. I. Simpson's proximal scaler No. 13) until the groove is about one-eighth of an inch deep. Always be sure to have the apex of the groove pointing away from the ridge, or rather where there ought to be one.

After packing with any rubber you choose to use, and just prior to closing the flask finally, cover the model with a generous amount of chloroform applied with a soft brush; then quickly cover the whole model with one thickness of palate or velum rubber. Be sure to get it over the grooves and work quickly before the chloroform evaporates.

I also use this method, instead of a vacuum chamber, for upper plates where the roof of the mouth is very hard. I outline a groove the shape of a vacuum chamber, or any shape the hard part of the roof of the mouth may indicate.

Try this and you will have a suction that only a rivet through upper jaw could beat. I give this method to my professional brethern for the good of many a dissatisfied patient, and am sure that it will save those who try it many sleepless nights, and, perhaps, some language that wouldn't look well in print.—H. J. Fenn, D.D.S., *Western Dental Journal.*

*Dangers of Chronic Alveolar Abscess.* It seems strange that dentists, physicians and laymen each view with complacence chronic alveolar abscess in the mouth, when, if such an abscess were observed in any other part of the osseous tissues, it would at once cause alarm and a remedy sought for its cure.

What are some of the results of chronic alveolar abscess? Large areas of bone, in proximity to diseased roots become destroyed by inflammatory processes, caries of bone is established which continues the destruction of the bone, the granulating surfaces absorb the tonic elements and systemic disturbances of more or less grave consequences may ensue. The more progressive internes are now looking into the mouths of their patients to discover if there may not be there found a cause for subtile phenomena not discoverable elsewhere, and they are not looking in vain. Recently Dr. Billings, of Chicago, had a patient with multiple neuritis, with partial paralysis of the lower limbs. He could find no reason for the condition until he explored the oral cavity; here he discovered in the maxilla an abscess. He ordered the teeth in the diseased area removed and the pus cavity curetted and drained. The condition was at once improved and in a comparatively short time the patient recovered. A guinea pig was inoculated with organisms from the abscess with a similar train of symptoms following. Another case, a physician who had been out of health for some time, came to Chicago from Wisconsin to consult a brother practitioner. He had a temperature slightly above normal and a painful synovitis of the knee joint. The latter was notof rheumatic or gonorrheal origin. Not finding a cause for the symptoms, he sent the patient to me for examination of the mouth. I found the upper left cuspid and first biscuspid pulpless, with a small sinus discharging pus. I had a radiograph of the affected bone area made and discovered a large abscess about the roots of these teeth. Two-thirds of the entire roots were fully uncovered, the bone having been destroyed. I removed the teeth and found their uncovered portions thoroughly necrosed. I curetted the pus cavity, drained, irrigated and packed. In a few days the temperature was normal, and in two weeks the synovitis disappeared and the patient wrote me that he felt practically cured with no other treatment. In order to effect this cure I sacrificed two most important teeth, but they were in a condition which no amount of treatment could have restored to health.

Many other cases might be cited indicating similar serious conditions resulting from like causes which, as in these cases, were unsuspected by the patient himself, since the abscess did not attract his attention. No abscess of the jaws should be permitted to continue indefinitely. It should be cured. If treatment for a reasonable time through the roots of the teeth does not effect a cure, then either the excision of the dead portion of the root or roots with curettment of the abscess walls should be done, or the extraction of the offending organ. The danger of systemic poisoning is too great to permit the condition to continue indefinitely. It may be that so long as the physical condition is up to par no evil results may come, but if the bodily vigor fails, nature may not be sufficiently strong to resist.—Dr. T. H. Gilmer in *Western Journal.*

*Abscess Remedy.*

℞

| | |
|---|---|
| Bichloride of Mercury | grs. vj |
| Phenol | ʒj |
| Sassafras Oil | gtts xy |
| Alcohol | ℥jv |

Mix, dilute with warm water to suit conditions and inject as a disinfectant irrigant.

W. H. WHITSLER, M. D., D. D. S.

*Disinfectant for Instruments.*

℞

| | |
|---|---|
| Mercuric Chloride | grs. 2½ |
| Tincture Green Soap | ℥j |

Mix and dilute with soft water to desired consistency.

J. W. JUNGMAN, D. D. S.

*Poison Ivy Remedy.*

℞

| | |
|---|---|
| Acetate of Lead | grs. x |
| Spirits of Nitrous Ether | ℥j |

Sig. Apply to affected parts.

*For Pain After Extraction.*

℞

Chloretone,

Campho-Phenique, to saturated solution.

Sig. Apply on pledget of cotton to socket.

L. A. JORDAN, D. D. S.

*The Effects of Tobacco on Body and Mind.* While it has been shown that nicotin acts as a poison in the lower animals, depressing the nervous, circulatory and respiratory systems, there is considerable diversity of opinion as to the effects of tobacco on human beings. There is, however, a fairly well established belief that even the moderate use of tobacco is not without possibilities of evil, while excessive use is distinctly harmful. The effects, naturally, are more pronounced in youth and adolescence than in later life. Some observations made by Dr. George L. Meylan, of Columbia University, are interesting in this connection. In a study of 223 college students from two classes it was found that 115 were smokers and 108 non-smokers. The smokers were a little older than the non-smokers, and they presented only corresponding differences in measurement (weight, height, lung capacity, total strength). On the other hand, the non-smokers exhibited a distinct advantage in scholarship, but in this connection other considerations must be taken into account. For one thing, the smokers participated in larger proportion than the non-smokers in athletics and in fraternity membership, and the scholarship records of smokers, athletes and fraternity members alike were lower than those of other students. Besides, there is a leisure class type of college students, who smoke and go in for athletics and fraternity membership, but who, as a rule, do not attain high grades of scholarship. The non-smoker, on the other hand, is usually ambitious, industrious and self-dependent, and with less inclination and opportunity for the athletic and social aspects of college life. There will be no serious dissent from the following conclusions: The use of tobacco by adolescents is injurious; there is no scientific evidence that the moderate use of tobacco by healthy mature men produces any beneficial or injurious physical effects that can be measured; there is an abundance of evidence that tobacco produces injurious effects in (a) certain individuals suffering from various nervous affections, (b) persons with an idiosyncrasy with respect to tobacco, (c) persons who use it excessively. It is generally conceded that the use of tobacco by college students is closely associated with idleness, lack of ambition, lack of application and low scholarship, though these may not be due entirely to the tobacco.—*A. M. A. Journal.*

*The Purification of Water by Storage.* It has long been known that when cholera vibrios or typhoid bacilli are added to raw river water under laboratory conditions of experiment thes pathogenic microbes die off rather rapidly; over 99 per cent. of the two species mentioned are destroyed within the first week. The inference seems justified that the longer a contaminated water can be kept from reaching the consumer the less likely is it to be the bearer of infection. The time element is probably more important than any other in the familiar process of the self purification of streams, and its effect is particularly marked when water is retained for any considerable period in storage reservoirs.

In some cases the principle of purification by storage has been recognized and taken advantage of by the officials in charge of municipal water-supplies, but, on the other hand, storage has been looked on often merely as a safeguard against draught and has not been utilized to its full value as a protection against water-borne infection.

This seems, for example, to have been until recently the case in London where, it is stated, the two large Staines reservoirs with a total capacity of 3,338 million gallons had their inlet and outlet pipes in the same towers, thus rendering impossible any proper circulation of the water. The work of Houston,[1] however, has drawn attention to the influence of storage on the character of the London supply, and should go far to make the beneficial effect of water storage more generally appreciated.

Briefly speaking, storage reduces the amount of suspended

1. Report of the Metropolitan Water Board, 1905-1910.

matter, the amount of "free ammonia" and of "oxygen absorbed" and removes some of the color; the hardness is also usually decreased. The number of bacteria of all sorts is diminished by storage, and this diminution is especially marked in case of the excremental bacteria, including the typical *Bacillus coli.* Indeed, Houston's work demonstrates that storage "reduces the number of typical *B. coli* to a proportionately greater extent than it reduces the number of bacteria of all sorts." As a prefiltration process it lengthens the life of the filter and produces uniformity in the character of the water delivered to the filtration beds.

The important fact is brought out also in the Report of the Metropolitan Water Board that London is not really drinking filtered raw river water, but river water which by storage processes has been bacterially purified to a remarkable extent antecedent to filtration. In spite, therefore, of the somewhat unsavory origin of much of the river water which serves as the source of the London supply, the water actually distributed to the consumers is of a high degree of bacterial purity. It seems very unlikely that any noteworthy portion of the typhoid fever in London at the present time is water-borne. At all events the death-rate from this disease is as low (3 per 100,000 inhabitants in 1909)! as in those cities that are provided with a supply of known and unexceptionable purity.

Curiously enough, sone of the English authorities now appear inclined to attribute more importance to storage than to filtration in the purification of polluted river water. It does not seem necessary, however, to place the two processes in antithesis. Sand filtration, even when a raw and rather highly contaminated water is directly applied to the filter bed, has abundantly demonstrated its efficiency as a means of reducing typhoid infection. But it is indeed true that the beneficial effects of storage need to be just as widely known; in many cases this important process of purification can be taken advantage of with safety and economy. —Ed.—*Journal A. M. A.*

*School Clinics.* The opposition offered but a few years ago to the establishment of Schlool Clinics seems latterly to have quite given way. Initial experiments having resulted in immediate benefit to the child and to school work, and fewer citizens being nowadays indifferent to prospective benefits for their children, the facts are prooving too strong for any resistance based on adherance to merely academic and somewhat vague "principles" of individualism, the objection to pauperization. The undermining of parental responsibility, and so on. Judging from the firmly expressed opinions of medical officers, and the present attitude of the Press and of the public mind, any fresh proposals for the institution of Dental Clinics are likely to be favorably received, and wherever the difficulties of expense can be got over, such clinics are certain to be established in many other towns.—*Dental Record.*

*Keep at School.* We are always preaching to the kids to make the most of the superior opportunities of the present time at school, and at the same time neglect to improve our own opportunities, which are just as superior as are the kids'. School never closes to the human race. This is especially pertinent to the dentist who now and again airs his grievances against the dental school he attended because it did not teach him as much as he now thinks it should have done. Doubtless the school gave him quite as much as his brain then permitted him to grasp. He has grown, he is able to take more, he is more appreciative, he feels his needs and is in a better condition to be a schoolboy than he was when on the anxious bench, with strained nerves waiting and fearing the expected little note from the faculty. The knowledge is his, all that he wants, by paying for it with a little brain work. Don't be a "hobo," constantly complaining that the world is slow in paying you the living it owes; don't be among those who are always wanting to divide with the fellow who has a little more. Mingle with your fellows, attend night school in a class of one, or a correspondence school, observe, read, and everlastingly work, work, and keep on working until you get all you want. If you are in earnest the undertaker will demand his fee before you are through, for to keep pace with this racing age no one can cease learning until ready to go under the sod.—W. H. Trueman, in *Brief*.

*Unhygienic Cigar-Making.* A matter worth the attention of health authorities is afforded by the custom among cigar-makers of biting off the tobacco at the end with their teeth and finishing the cigar by plastering down the outside leaves with saliva. We cannot say how extensive this custom is, but from our acquaintance with cigar-makers it appears not to be uncommon. The excuse given for this insanitary method is that, while the managers do not actually demand it, it is practically impossible to finish a cigar to meet the demands of the foremen without resorting to it. The practice if generally known ought to diminish the sale of cigars on esthetic considerations alone, to say nothing of the very obvious danger of the transmission of disease—tuberculosis, for example, which is quite prevalent among cigar-makers. The practice is injurious not only to the customer, but to the workman as well. The frequent occurrence of functional stomach disease, among them, chiefly hyperchlorhydria, is at least greatly favored by this practice.—*Journal A. M. A.* September 17, 1910.

*Non-Enforcement of Anti-Spitting Ordinances.* From information recently collected it appears that out of seventy-four cities reported as having anti-spitting ordinances only thirty-six had made any arrests. It is found that in the enforcement of these ordinances health officers are somewhat more vigilant than the regular police. Newton (*Journal of the Outdoor life*, August, 1910) says that the real reason for the non-enforcement of the ordinance is indifference on the part of the citizens, lack of civic pride and failure to appreciate the danger from the unrestrained practice of the habit. He says that the police fail to enforce it as they regard it as merely intended to abate a minor nuisance, but that health officers are primarily responsible, as the community looks to them for leadership in all health matters. It seems to require specially detailed officers or inspectors. It is probable, however, that the public agitation and the numerous means of calling attention to the danger have led to a very decided abatement of the nuisance.—*A. M. A. Journal.*

*The Oral Cavity of Maternity Nurses—A Dangerous Source of Infection for their Patients.*

As is well known, the genital organs of a lying in woman offer an exceptionally favorable field for infection by pathogenic bacteria, especially streptococci and staphylococci, and for centuries puerperal fever has been one of the most dreaded complications of childbirth. Owing to the wonderful progress of prophylaxis, the rate of mortality has been reduced in maternity hospitals to 0.1 per cent., in private cases to from 0.3 to 0.4 per cent. Nevertheless, in Prussia alone from 4,000 to 5,000 cases of puerperal fever per annum terminate fatally; the most rigorous regulations regarding antiseptic measures are therefore being enforced among maternity nurses,—yet one very dangerous source of infection, the oral cavity, has been unduly overlooked. Recognizing this lamentable fact, the author, in spite of the great difficulties encountered, secured permission to examine the mouths of the nurses in the provincial schools for nurses of Breslau and Oppeln, with the following results: In thirty-five nurses examined who bad been in practice for some time the following conditions were noted: Number of teeth present, 376; carious teeth, 191; roots present,234; artificial teeth, 19; gingival fistulae, 7; tartar and plaques, 26. The oral conditions noted in thirty-six nurses still in training were even more appalling. It is easily seen how great the possibility of infection is by transmission of streptococci and staphylococci from the mouth of the nurse to the mother, either by expiration or manual contact. Experiments in which cultures were made from agar slabs against which the nurses had breathed from distances of 15 and 30 cm. before and after their mouths had been put in hygienic condition, also from the scrapings from under the disinfected finger-nails before and after the hand had been in contact with the mouth before and after sanitation, showed that inumerable micro-organism, among which, besides various other pathogenic germs, may be found streptococci and staphylococci, are transmitted from the nurse to the patient by expiration and manual contact, and that careful hygiene of the mouth reduces the danger of such infection to a uniformly small degree. On the basis of these findings, the author makes the timely plea that the following rules for nurses, both in practice and in training be adopted and rigorously enforced:

(1) Every woman on being admitted to a school for nurses, besides a medical certificate as to her general state of health, must produce a dental certificate attesting to the healthy or hygienic condition of her mouth.

(2) During her training the nurse must be instructed in the importance of oral hygiene and in the rational care of the mouth and teeth. She must especially be enlightened in regard to the dangers arising to maternity patients from any neglect of oral hygiene on her part.

(3) Every year the maternity nurse must submit to the head physician a certificate from a specially appointed dentist attesting the healthy condition of her mouth.

(4) The nurse is obliged to continually devote her fullest attention to the care of her mouth and teeth, and, especially before taking charge of a case, to carefully cleanse and disinfect her mouth.

(5) The text-books for nurses should contain a chapter on dental and oral hygiene, and the chapters on suppuration should also contain references to suppurations in the mouth, especially in connection with carious and ulcerated teeth and roots.—Dr. Guttmann, in *Deutsche Monatschrift.*

Dr. Guttmann's essay demonstrates most forcibly the significance of oral hygiene in a specially important field.

*First Permanent Molars: Their Treatment.* In cases of extensive decalcification of the first permanent molars without pulp involvement, copper, cement or zinc phosphate makes a desirable filling. For fear of operative exposure all decalcified dentin need not be excavated; after the cavity has been excavated as far as seems prudent, it is saturated with phenol, dried, then treated with silver nitrate and filled with cement. Later, when this cement has worn, it may be covered with alloy with far more safety than had the alloy been used at first. Should pulp exposure exist and capping be deemed prudent, the capping material used should be non-irritating, germicidal, with some lasting antiseptic properties, and have body. Such a combination we have in a paste of phenol, iodoform and zinc oxid. Cement is the best filling over this cap. It can be applied without pressure, and as its durability is limited, when refilling is indicated, opportunity is given to see the result of the treatment. All pulp capping may be regarded at best as tentative. It may not be permanently successful, and yet successful in this—it has allowed the natural process of growth to materially improve the tooth and add to its permanent usefulness. The operation may result in any one of three possible results. The pulp infection may have been so great that it is not affected by the treatment, and pain within a few days after the operation will require the removal of the filling and the adoption of further procedures; structural change of the pulp from the exposure may prevent repair, then the pulp slowly dies without pain, and as a rule, without suppuration during the life of a cement filling; in rare cases the pulp may continue vital and normal under this treatment, sealing the exposure with calcific deposits.—Dr. P. B. McCullough, Philadelphia, Pa.

*Professional Elevation: How to Attain.* I have been surprised at the number of papers which have been read by members of our profession which had for their text, "How best can we elevate the standing of our profession?" Nearly every paper points out that it must be done by the advancement of fees. This argument appears to me to be utterly absurd I am thoroughly convinced of the fact that a man should endeavor to legitimately increase his income, but let us for once and all try to rise above the idea that a man's worth is measured by his money. The average dentist has a far larger income than the average teacher or minister, and I think I may safely include in this the average physician or lawyer. Then why harp on this line any longer? The dental profession is honored and elevated by having as one of its members such a man as was the late Dr. William Norman Brodie, one of the greatest entomologists in America, a biologist well known to the scientific world, and yet almost unknown to his fellow practitioners. He did not live in a fashionable quarter, nor did he charge high fees, but let me say that he was loved and revered by many who knew him as one who possessed a master mind. His unselfish career, his honest work as a dentist, his scientific attainments are an honor to his profession, and the dental profession will be honored, elevated, and recognized as a noble profession just as its members recognize and honor their meritorious fellows.—Dr. M. P. Corrigan, *Dominion Dental Journal*, October, 1909.

*Hard Gums.* The thing that presents itself most forcibly to me is the lack of knowledge in parents of the laws of physiology. That is, the lack of proper knowledge of the conditions under which to bring up children, and from our view-point the mouth is very important. I have read in various works that the saliva of animals is strongly alkaline, but to go to the abattoir, apply a bit of litmus paper to the saliva of animals and find it change as quickly from acid to alkaline as it would be in a solution of bicarbonate of soda, and find this in absolutely every case, is something that should be considered by us. I take it that civilized environment has changed the physiology of man to almost the opposite condition. For it is a rare thing to find human saliva more than barely alkaline. Now, with the strong alkaline condition found in the mouths of animals, no acid decay could exist. Again, the eating of the rough food by these animals constantly rubbing against their gums, keeps the tissues healthy and strong. This feature is of great importance for there is a development of large rolls of connective tissue or callus at the necks of the teeth of these animals, especially just inside of the lower front teeth, where the food comes in contact with the mucous membrane. This is true in carnivora, where the teeth are conical, but not to the degree found in the herbivora; the biting with conical teeth also cleans them to the gums. When animals are kept from their normal diet and fed on civilized food, there is a marked delicacy or softening of the gums in consequence. I do not believe these points have been brought before our present generation strongly enough to impress them properly, and, since our teeth are not cleansed by our food, and our mouths do not have this protective alkaline saliva, by all means let us use the superior intelligence Providence has given us and give our gums this hard rubbing that they need, and the mouth and tongue the proper cleansing and the result will be to the development of healthy, strong gums and good teeth. Our intelligence should lead us to know where to begin and how to correct these defects; then we will know how to teach our children about them.—Dr. M. H. Fletcher, Cincinnati, in *A. M. A. Journal,* October 1, 1910.

